# The Old Paths

**Published:** 1871-03

**Authors:** 


### "THE OLD PATHS."

An admiring correspondent writes: "I have been reading with interest three volumes recently published by you, and my confidence in the medical profession is a little strengthened, for I confess it was utterly destroyed. You invariably advise the use of medicines under the eye of an educated physician; but, sir, the old practitioners are all dead, and alas for their successors, there are none in our region."

The medical profession — we mean the old school, allopathic, calomel-giving doctors, we acknowledge no others — has for a number of years been losing its preëminence, first, from lowering the standard of proficiency; second, from yielding to the clamor of a class of men who are low in their origin, vulgar in their tastes, unprincipled as to morals, and uncultured as to mind, at the same time ignorant and specious.

There is virtue in all classes of practitioners; there are advantages in the Water-cure treatment, in Homeopathy, in Vegetarianism, in Eclecticism, when practiced by men of education and high moral principle, but with honorable exceptions here and there, the doctors of these schools are ignorant, uncultivated, and unprincipled men. They murder their hundreds every year with no better ground to stand upon than Spiritualists, Magnetizers, and Electricians, who pocket their millions from gullible and weak-minded patients.

The regular medical profession are at fault in yielding to the clamor of these outsiders; they seem in many cases to be actually afraid to give a dose of calomel unless in a surreptitious way. The water-cure men are constantly inveighing against the use of drugs, and shamelessly manufacture cases of individuals who, as they assert, have been "drugged to death." Their teachings have not only spread among the common people, but seem to have had an effect on medical men themselves, causing them to speak and act as if they wanted to "curry favor" with those who have consulted them, by disparaging the use of medicine. A man came to me not long ago, having just consulted one of the professors in one of the regular medical colleges in New York City, alleging that the last advice given by the professor was, "Whatever you do, do not allow any doctor to persuade you to take medicine." Such a statement, if made, was certainly "unprofessional," and, besides that, was cowardly and exceedingly imprudent. The wiser and more generous course would have been to say, "I may be mistaken in my views, but if you consult any other physician, do him the justice to follow his advice."

Our own established convictions, increased with increasing observation, are, that medicine, wisely given, will hasten restoration to health, and save multitudes from a premature grave. The great misfortune of the age and of the medical profession is that the people do largely take medicine on their own responsibility, and only come to the physician when they have so drugged themselves, and lost so much time, that it is too late to be saved; and the reader is earnestly counselled to this one plain, rational course, — if you are sick or suffering, either do nothing but keep warm in bed in a well-aired room until you are better, or send at once to a regular educated physician of the old school. To do differently, is to act unwisely.

